# Liver Transplantation for Hepatitis C and Alcoholic Liver Disease

**DOI:** 10.1155/2010/893893

**Published:** 2010-12-15

**Authors:** Marco Carbone, James Neuberger

**Affiliations:** Liver Unit, Queen Elizabeth Hospital, Birmingham B152TH, UK

## Abstract

End-stage liver disease due to hepatitis C (HCV) and cirrhosis from alcohol (ALD) are the commonest indications for liver transplantation in the western countries. Up to one third of HCV-infected transplant candidates have a history of significant alcohol intake prior to transplantation. However, there are few data available about the possible interaction between alcohol and HCV in the post-transplant setting. Patients with both HCV and alcohol are more likely to die on the waiting list than those with ALD and HCV alone. However, after transplantation, non-risk adjusted graft and patient survival of patients with HCV + ALD are comparable to those of patients with HCV cirrhosis or ALD cirrhosis alone. In the short and medium term HCV recurrence after transplant in patients with HCV + ALD cirrhosis does not seem more aggressive than that in patients with HCV cirrhosis alone. A relapse in alcohol consumption in patients with HCV + ALD cirrhosis does not have a major impact on graft survival. The evidence shows that, as is currently practiced, HCV + ALD as an appropriate indication for liver transplantation. However, these data are based on retrospective analyses with relatively short follow-up so the conclusions must be treated with caution.

## 1. Background

In the Western world, the commonest indications for liver transplantation are end-stage cirrhosis due to hepatitis C virus (HCV) infection and, secondly, cirrhosis from alcohol (ALD) [1]. The medium and longer term outcome of patients undergoing transplantation for HCV-related cirrhosis is reduced because of the impact of recurrence of hepatitis C: this occurs almost invariably for those who have HCV RNA prior to transplant. 

Early reports suggested that HCV recurrence was a relatively benign condition after liver transplantation [2–4] but in 1999, Forman et al. [1], using the United Network for Organ Sharing (UNOS) database of patients grafted between 1992 and 1998, showed that HCV-infected patients had worse patient and graft survival than those with chronic cholestatic diseases, a similar outcome to those transplanted for hepatitis B infection, autoimmune hepatitis, cryptogenic cirrhosis, and ALD and better than that in patients undergoing transplantation for cancer. An analysis of the UNOS database of patients grafted between 1990 and 1996 by Roberts et al. [5] reached similar conclusion. In a relatively large proportion of cases, HCV recurrence has an aggressive course, with progression to cirrhosis within 5 years, resulting in survival rates of less than 65% [6–8]. Treatment of the HCV is, with current treatments, relatively ineffective and appears to be more toxic than in the native liver.

Alcoholic liver disease (ALD) is, for selected patients, an excellent indication for liver transplantation, with outcomes at least as good as for other indications and with a rate of alcohol relapse acceptably low [9–11]. Nonetheless, it remains a controversial indication for liver transplantation, due to concerns that in some cases, the recipient will return to a pattern of alcohol consumption, leading to graft failure, noncompliance, or other potentially fatal complications [12].

Many patients with alcoholic liver disease have associated hepatitis C viral (HCV) infection, and, conversely, many people, infected with HCV drink above the recommended limits for alcohol. There is mounting evidence that alcohol abuse may accelerate the course of chronic hepatitis C [13–18]. However, there are few data available about the possible interaction between alcohol and HCV on the posttransplant setting. Up to one third of HCV-infected transplant candidates have a history of significant alcohol intake prior to transplantation [19], but the drinking habits in HCV cirrhotic patients are often not always well explored. This probably has the effect of significantly underestimating the impact of alcohol in causing end-stage liver disease and overemphasizing the serious consequences of HCV infection. Furthermore, after transplantation, patients with HCV infection (whether or not associated with alcohol) tend not to be monitored as closely for alcohol consumption as those with HCV infection. The effect of other agents, such as use of illegal drugs (such as cannabis), is also not well studied even though such agents are associated with accelerated progression of HCV.

In this paper, we review the current evidence on the effect of HCV and alcohol use on the listing and outcome after liver transplantation, compared to those with HCV or alcohol alone, as a cause of the liver failure.

## 2. Is the Outcome of Liver Transplantation in Patients With HCV + ALD Similar to Those Grafted for HCV or ALD Alone?

Current evidence supports HCV + ALD as an appropriate indication for liver transplantation, as highlighted by several studies suggesting that patients with HCV + ALD have similar graft and patient survival outcomes as patients who undergo transplantation for HCV or ALD alone [20–25] (Table 1).

In an early study, Dhar et al. in 1999 [20] showed little difference in patients and graft survival between patients undergoing transplantation for HCV alone or for HCV infection plus alcohol. However the period of followup was relatively short (mean 29 months). Subsequently, several other early studies [21–23] have analyzed the consequences of HCV infection in patients with ALD undergoing OLT, and they found that pretransplant HCV infection in ALD patients does not affect the survival after OLT but these series included only small numbers of patients and followup was relatively short. Recently, Aguilera et al. [24] analyzed retrospectively the clinical and histological outcome of 60 patients undergoing liver transplantation for cirrhosis of mixed etiology (HCV and alcohol) and compared it with that of patients undergoing liver transplantation for HCV-related cirrhosis (*n* = 170) or alcohol-related cirrhosis (*n* = 107). They found that patients transplanted for HCV + ALD had a better survival compared with patients with HCV alone, and similar survival compared with patients with alcohol alone. Patient survival at 1, 5, and 7 years was 86%, 73%, and 63%, respectively, in the mixed group, 72%, 49%, and 43%, respectively, in the HCV group, and 90%, 76%, and 67%, respectively, in the alcohol group (HCV group versus mixed group, *P* = .0001; mixed group versus alcohol group, *P* = .74). Graft survival was significantly lower in patients with HCV-related cirrhosis compared to the other two groups. The rate of retransplantation was higher in the mixed group, and the main cause of retransplantation in this group was viral recurrence. The main causes of death in the mixed group were recurrence of hepatitis C and complications possibly related to excessive immunosuppression such as sepsis. No difference were found in the incidence of severe recurrent HCV disease or fibrosis stage >1 at 1 year between mixed group and HCV group. The authors suggest that the better survival of the mixed group might be explained by two factors: the greater use of antiviral treatment in the mixed group versus the HCV group, because patients belonging to the former group were younger and so more likely to receive an antiviral treatment, and the young age is itself consistently associated with better survival. In the mixed group, alcohol was assumed to contribute mainly to the progression of liver disease prior to transplantation. After transplantation, when “alcohol factor” disappeared in the majority of patients, the liver damage could be related only to recurrent hepatitis C, similar to that occurring in patients without a history of alcohol intake prior to transplantation; this would explain the similar histologic HCV-progression in both groups in this study. 

The cumulative experience of multiple European transplant centers have confirmed that the survival rates following liver transplantation in the European liver registries are similar for patients with ALD and ALD plus viral etiology (HCV and HBV), but patients with ALD plus HCV had significant lower survival compared to ALD plus HBV infection [25]. 

Results show that concomitant infection with HCV eliminates the progressive improvement of survival over time in ALD patients. An adverse role of HCV coinfection in patients transplanted for ALD liver cirrhosis is in keeping with the finding of more aggressive liver damage was in ALD plus HCV infected patients. In the same study, the authors found that de novo tumors were a major cause of death in the ALD group, but not in the HCV + ALD group and HCV group (13.7%, 8%, and 5%, resp.). Patients transplanted for ALD with and without associated HCV infection had a significantly higher incidence of death due to cardiovascular events compared to patients with HCV alone (7.4%, 8%, and 5.3%, resp.). Also, patient with ALD and ALD + HCV had a greater incidence of deaths caused by social problems, including suicide, compared to patients with HCV alone (1.3%, 1.2%, and 0.6%, resp.) [25].

Interpretation of findings from registries will inevitably have limitations: the data may be incomplete and definitions and protocols for selection, transplantation, and followup vary between participating centers. Furthermore, case mix (as discussed below) may differ between the groups. Nonetheless, that the two large registries provide similar outcomes implies that the conclusions are valid.

Thus, for those transplanted for HCV, it is important that the recipient and the clinician are aware of the importance of limiting alcohol consumption in order to prolong patient and graft survival. 

## 3. Is There Any Difference in the Baseline Characteristics of Transplanted Population with Both Hepatitis C and Alcoholism Compared with Patients with HCV or Alcoholism Alone?

HCV + ALD patients undergoing liver transplantation are usually younger than those transplanted for hepatitis C or alcoholic liver disease alone [20, 24, 25], in keeping with other evidence that hepatitis C and alcohol act synergistically to cause more aggressive liver disease. Goldar-Najafi et al. [23] suggested a difference in pre-OLT duration of liver disease between those grafted for ALD alone compared with those grafted for HCV + ALD (median 25 and 15 years, resp., but the strength of the conclusions is limited by the numbers of cases in each groups).

Similarly, Aguilera et al. [24] found that patients with alcoholic cirrhosis were sicker at the time of transplantation than those of the HCV and HCV + ALD groups (percentage of patients with Child-Pugh-Turcotte C: 47%, 30%, and 43%, resp.; mixed versus ALD, *P* = .01). A history of tobacco consumption was more frequently reported in patients undergoing transplantation for HCV + ALD and ALD alone than those with HCV alone (72%, 68%, and 24%, resp.; HCV versus mixed, *P* = .001). The incidence of HCC pre-OLT was greater in HCV and HCV + ALD patients than in ALD patients (44%, 35%, and 18%, resp.; mixed versus ALD, *P* = .01). Similar findings have been reported by others authors [22, 23]. Goldar-Najafi et al. [23] reported that the tumors in the mixed group were larger than those in the ALD group (mean diameter 4.25 versus 0.85 cm). Post-OLT recurrence of HCC was noted in 25% in ALD compared with 50% in those with HCV + ALD, suggesting the recurrence rate is related to their size of the tumour at LT rather than any effect of the coexistent HCV (Table 2).

While there may be some element of selection bias and different patterns of referral for those in the three groups, those transplanted for ALD and HCV are more likely to have more aggressive disease pretransplant and have more advanced disease at the time of transplant.

## 4. Is the HCV Recurrence More Aggressive in the HCV-ALD Transplant Patients?

Several studies pointed to a link between alcohol and progression of liver disease due to viral hepatitis. Alcohol will transiently increase circulating levels of HCV RNA, suggesting that alcohol may have a direct effect on viral replication (26). Patients with HCV infection who have high alcohol consumption have higher hepatic iron concentrations, which may affect HCV replication and also perhaps enhance hepatic fibrogenesis (17). Moreover, the immunologic effects of alcohol may affect the balance between host and virus, resulting in more severe liver disease.

However, there is, as yet, no convincing evidence that HCV recurrence in patients transplanted for HCV + ALD cirrhosis is more aggressive than that in patients with HCV cirrhosis alone [23, 24]. 

No differences were found in the incidence of severe recurrent HCV disease (45% in the HCV group versus 45% in the mixed group, *P* < .660), acute hepatitis (26% in the HCV group versus 28% in the mixed group, *P* = .854), or fibrosis stage >1 at 1 year (34% in the HCV group versus 35% in the mixed group, *P* = .88) between patients undergoing transplantation for HCV-related cirrhosis alone compared to those with cirrhosis of mixed etiology [24]. 

However, these conclusions must be treated with caution: numbers are relatively small and many factors (host, viral, graft, and immunosuppression) may affect rates of rate recurrence.

## 5. Does Alcohol Recidivism Represent an Important Issue in Transplanted Patients for HCV-ALD Cirrhosis?

The risk of recidivism is a major problem in alcoholic cirrhotic patients although the actual incidence is difficult to establish as centers use different evaluation methods [26–29], definitions of alcohol recurrence, and different followup protocols. The effect of alcohol recidivism in terms of histological liver damage has been reported in several studies [30–35], with conflicting results in terms of severity [30, 32, 33, 35, 36]. Determining the contribution of alcohol and HCV infection to the extent of graft damage can be difficult [22, 37, 38]. There is no clear definition of relapse, and this lack of definition may explain the widely different relapse rates reported in the literature, ranging from 7% to 95% [35, 39, 40].

Cuadrado et al. showed that heavy drinking reduces the long-term survival (over 5 years) of patients transplanted for ALD alone [41]. However, the significance of slips remains uncertain: it has been hypothesized that a slip, which should always be considered an unwelcome event but, in itself is unlikely to cause harm but, for many patients will progress to a more damaging pattern of alcohol intake [42]. Thus, most centers advocate total abstinence in those with a history of alcohol dependence.

Goldar-Najafi et al. reported a similar rate of relapse to alcoholic behaviour in ALD and HCV + ALD groups (8,9% versus 9,4%, resp.), but overall return to heavy drinking seemed to be uncommon [23]. Aguilera et al. found that the incidence of alcohol relapse was higher in patients undergoing transplantation for ALD than in the HCV and HCV + ALD groups (18%, 3%, and 8%, resp.) [24]. Burra et al. [21] studied the effect of alcohol recidivism in terms of liver histopathological damage in patients following liver transplantation for alcoholic cirrhosis, with or without concomitant HCV infection. They classified the patients as heavy drinkers if they drank more than 200 g of alcohol a week and occasional drinkers if they drank less than 200 g of alcohol a week [33]. No statistically significant difference emerged between HCV positive heavy drinkers, occasional drinkers, and abstainers in terms of fatty changes in the hepatocytes, pericellular fibrosis, and perivenular fibrosis. Portal tract fibrosis (*P* = .0005), portal tract monocyte (*P* = .003) and lymphocyte (*P* = .001) inflammation, parenchymal lymphocyte inflammation (*P* = .006), and perivenular fibrosis (*P* = .025) were more common in anti-HCV positive compared with HCV-negative patients, suggesting graft damage due to the virus damage rather than with alcohol consumption. However, because of the relative low rate and short duration of alcohol use following liver transplantation, these conclusions must be treated with caution. No significant differences in liver tests were seen at any time after liver transplantation among the three groups of drinkers with or without HCV. It seems that the majority of patients who resume drinking habit after liver transplant consume moderate amounts of alcohol (fewer than 12% of patients drink >200 g of alcohol/week), and therefore such liver damage is slow to develop. 

On the present, limited information, alcohol recidivism after liver transplantation in patients with HCV + ALD cirrhosis does not appear adversely to influence liver morphology or function. The predictors of recidivism of alcoholism are uncertain [35, 43]. The role of recurrent HCV in inducing the alcohol behaviour or potentiating the injury is unclear.

## 6. Is the Waiting List Mortality and Posttransplant Mortality Increased in Patients Transplanted for HCV and Alcoholic Cirrhosis Compared with Alcohol or HCV Infection Alone?

HCV patients may be at increased risk for waiting list mortality when compared with patients without HCV, and this effect is greater in subjects with both HCV and ALD [44]. One study from the US showed that for those with HCV infection, there was a significantly greater risk of death on the waiting list (HR 1.19, 95% CI 1.09–1030, *P* = .001) whereas for those with ALD there was no significant increased risk of death. However, of those with HCV infection, those with ALD had a greater risk of waiting list death than those with HCV alone (HR 1.14, 95% CI 1.04–1.25, *P* = .006) and conversely for those with a primary diagnosis of ALD, those with HCV had a greater risk of waiting list mortality than those with ALD alone (HR 1.36, 95% CI 1.21–1.53, *P* < .0001). Thus, while it may be difficult to distinguish whether ALD or HCV is the main cause of end-stage liver disease, it is clear that those with both HCV and ALD are more likely to die awaiting transplant.

Unadjusted and adjusted posttransplant mortality is greater in HCV+ subjects than HCV− recipients (HR 1.26, 95% CI 1.10–1.45; *P* = .0009) whereas ALD did not influence posttransplant mortality (HR 0.95). ALD did not significantly contribute to mortality in either HCV+ or HCV− subjects. In contrast, HCV infection increase posttransplant mortality in both ALD + HCV recipients (HR 1.30, 95% CI 1.07–1.59; *P* < .01) and ALD alone recipients (HR 1.25, 95% CI 1.08–1.45; *P* = .004), with the interaction between HCV and ALD on posttransplantation mortality being nonsignificant (Table 3). Thus, while alcohol consumption does lead to graft loss or patient death in some recipients, the effect is too small to show statistical significance so the conclusion is likely to represent a type 2 error rather than support the view that a return to alcohol consumption has no impact on patient or graft survival.

The apparent lack of impact of alcohol may be due to low rate of posttransplantation heavy drinking habit relapse and to the fact that it takes up to 10 years to have an impact on graft [45–47].

Both ALD and HCV patients showed a survival benefit from liver transplantation. The survival benefit of transplanted patient for mixed etiology has not been well evaluated although it is likely to be significant. 

## 7. Is There Any Difference in Terms of Quality of Life (QOL) among Patients Transplanted for HCV + ALD Cirrhosis Compared with Patients Transplanted for HCV Cirrhosis Alone?

Quality of life (QOL) is an important factor in the evaluation of OLT. The role of HCV infection on QOL and specifically the effect of recurrence of the infection after LT has been the focus of several studies [48–55]. In comparison with other OLT indications, hepatitis C report much lower health-related quality of life (HRQOL) scores after transplantation despite similar survival rates [49, 51, 53, 56, 57]; moreover, patient knowledge of the diagnosis of recurrent HCV alone can negatively impact HRQOL [48, 57–61].

Singh et al. evaluated prior alcohol use and the HRQOL scores of HCV transplanted patients. They found that Karnofsky scores at 6 months (mean, 82 versus 97) and 12 months (mean, 84 vsersus 85.7), the Beck depression scores at 6 months (mean, 13.4 vsrsus 14.9) and 12 months (mean, 19.5 versus 21.5), and quality of life scores at 6 months (mean, 5.0 versus 4.6) and 12 months (mean, 3.5 versus 2.6) did not differ significantly between patients with HCV recurrence and alcohol use and patients with HCV recurrence without alcohol use [49]. Multiple studies confirm these findings and show no significant difference in HRQOL with alcoholic liver disease as OLT indication compared with other OLT indications, including HCV [62–66]. Recently, Ruppert et al. reported that recipients who had both ALD + HVC, but not either diagnosis alone, had consistently worse QOL in all domains evaluated (physical distress, psychological distress, social/role function, personal function, and general health perception) at the 1-year time point, and they significantly worsen in both physical functioning and physical symptoms over time. These differences remain after controlling for other medical factors (age, MELD scores, etc.) before LT [67].

## 8. Conclusions

Current evidence suggests that Hepatitis C/Alcohol-related cirrhosis represents an excellent indication for liver transplantation, and that graft and patient survival are comparable to those of patients with HCV cirrhosis or ALD cirrhosis alone. HCV recurrence in patients with HCV + ALD cirrhosis undergoing liver transplantation does not seem more aggressive than that in patients with HCV cirrhosis alone. A return to alcohol consumption, in the short term at least in patients with HCV + ALD cirrhosis does not have an important impact on graft survival in patients with alcohol and HCV infection. However, outcomes are relatively short and, given the known interaction of HCV and alcohol on exacerbation of liver damage in the native liver, it would seem prudent to advise those grafted for HCV infection to have a minimal alcohol consumption, and where there is a history of alcohol dependence, complete abstinence should be advised.

## Figures and Tables

**Table 1:** Patient survival after liver transplantation.

Diagnosis	1-year survival (%)	No. of patients	3-year survival (%)	No. of patients	5-year survival (%)	No. of patients	10-year survival (%)	No. of patients
Hepatitis C								
Burra et al. [25]	81	4166			67	1906	54	475
Aguilera et al. [24]	72	68			49	83		
Dhar et al. [20]	80	25						

Alcoholic Liver Disease								
Burra et al. [25]	84	6301			73	2867	58	663
Aguilera et al. [24]	90	96			76	81		
Burra et al. [21]			64	33				
Dhar et al. [20]	90	22						
Pera et al. [22]	83	29						
Goldar-Najafi et al. [23]	93	52						

Hepatitis C + Alcoholic Liver Disease								
Burra et al. [25]	84	714			65	261	52	57
Aguilera et al. [24]	86	51			73	43		
Burra et al. [21]			82	16				
Dhar et al. [20]	72	7						
Pera et al. [22]	88	21						
Goldar-Najafi et al. [23]	97	31						

**Table 2:** Baseline pretransplant characteristics by group.

	HCV group	HCV + alcohol group	Alcohol group
Age (years)	50 [20]; 59 [24]; 53 [25]	43 [20]; 48 [22]; 46 [23]; 49 [24]; 49 [25]	51 [20]; 45 [22]; 53 [23]; 53 [24]; 52 [25]
Gender (% men)	51 [20]; 51 [24]; 63 [25]	70 [20]; 79 [22]; 91 [23]; 97 [24]; 88 [25]	81 [20]; 88 [22]; 84 [23]; 87 [24]; 79 [25]
Etnicity			
Caucasian (%)	81 [20]	73 [20]	92 [20]
Black (%)	13 [20]	27 [20]	4 [20]
Others (%)	6 [20]	0 [20]	4 [20]
Child-Turcotte-Pugh C (%)	30 [24]	58 [22]; 43 [24]	52 [22]; 47 [24]
Child-Turcotte-Pugh score	8 (5–13) [24]	9 (5–14) [24]	9 (5–13) [24]
HCC (%)	10 [20]; 44 [24]	0 [20]; 6 [21]; 21 [22]; 35 [24]	0 [20]; 3 [21]; 3 [22]; 18 [24]
History of tobacco			
Consumption (%)	24 [24]	72 [24]	68 [24]
Alcohol intake			
0–40 g/day (%)	61 [20]	0 [20]	0 [20]
40–80 g/day (%)	39 [20]	0 [20]	0 [20]
>80 g/day (%)	0 [20]	100 [20]	100 [20]
Genotype 1 (%)	92 [24]	81 [24]	
Pre-OLT duration of the liver disease in years (median)	—	15 [23]	25 [23]
Time on waiting list (days)	56 [24]	52 [24]	87 [24]

**Table 3:** Influence of diagnosis on pre- and posttransplant mortality in patients with hepatitis C+ alcoholic cirrhosis.

	Pretransplant	Posttransplant
Alcohol	HR 1.14	HR 0.97
	95% CI 1.04–1.25	95% CI 0.83–1.13
	*P* = .006	*P* = .70

Hepatitis C	HR 1.36	HR 1.3
	95% CI 1.21–1.35	95% CI 1.07–1.59
	*P* = .0001	*P* = .01

Data from Lucey et al. [44].
